# *Francisella tularensis* Subspecies *holarctica* in Stranded Beluga Whales, Cook Inlet, Alaska, USA

**DOI:** 10.3201/eid3106.250033

**Published:** 2025-06

**Authors:** Natalie Rouse, Jeremy Buttler, Kristy Pabilonia, Christina Weller, Laurel Respicio-Kingry, Elizabeth Dietrich, Jeannine Petersen, Ganna Kovalenko, Eric Bortz, Kathy Burek Huntington

**Affiliations:** Alaska Veterinary Pathology Services, Eagle River, Alaska, USA (N. Rouse, K. Burek Huntington); University of Alaska Anchorage, Anchorage, Alaska, USA (N. Rouse, J. Buttler, G. Kovalenko, E. Bortz); Colorado State University, Fort Collins, Colorado, USA (K. Pabilonia, C. Weller); Centers for Disease Control and Prevention, Fort Collins (L. Respicio-Kingry, E. Dietrich, J. Petersen)

**Keywords:** tularemia, bacteria, Francisella tularensis, holarctica, beluga whale, marine mammal, cetacean, metagenomics, MLST, nanopore, Alaska, United States

## Abstract

We report fatal tularemia in stranded beluga whales in Cook Inlet, Alaska, USA. *Francisella tularensis* was detected by nanopore metagenomics, confirmed by quantitative PCR and immunohistochemistry, and characterized as *F. tularensis* subspecies *holarctica* by multilocus sequence typing. Our findings should be considered when assessing biosecurity and marine mammal health in the North Pacific.

*Francisella tularensis* is a highly pathogenic gram-negative bacterium that infects a large range of animals and humans, primarily in the Northern Hemisphere, causing the clinical disease tularemia. Human disease manifests with influenza-like symptoms (lymphadenopathy, conjunctivitis, pneumonia, septicemia) and other specific symptoms corresponding to the route of exposure. Two subspecies, *F. tularensis* subsp. *tularensis* and *holarctica*, are known pathogens and can be acquired via multiple routes, including arthropod vector, cutaneous, ingestion, or inhalation (*1*).

*F. tularensis* was first documented in Alaska, USA, in 1938 (*2*) and has been isolated infrequently in ticks, lagomorphs, and rodents. Serologic studies have confirmed exposure in humans, avian species, terrestrial mammals, and polar bears in multiple areas of the state (*2*). In October 2023, tularemia was diagnosed in a pinniped in Washington, USA, when a biologist was infected during necropsy (*3*). The same fall, dead stranded beluga whales (*Delphinapterus leucas*) in Cook Inlet, Alaska, were found to have gross lesions consistent with tularemia. We report the results of an investigation of those deaths.

Necropsies were performed and tissues collected and stored following standard procedures. Samples for histopathology were fixed in 10% neutral buffered formalin (Table). We submitted varied tissues from 2 sufficiently fresh animals (no. 2023279: fetal spleen, mediastinal lymph node, spleen, blowhole swab, heart, liver; and no. 2023288: brain, liver, mammary gland, mediastinal lymph node, spleen) for aerobic culture and testing for known cetacean pathogens, including influenza and *Erysipelothrix* sp. by PCR, and for harmful algal bloom toxins by ELISA (Table). We analyzed blowhole swab, lung, mediastinal lymph node, and rectal swab samples from animal 2023279 by metagenomic sequencing. In brief, we extracted and amplified total nucleic acids (I.M. Claro et al., unpub. data, https://doi.org/10.12688/wellcomeopenres.17170.2) and sequenced cDNA and metagenomics libraries by SMART9N using an Oxford Nanopore Rapid PCR barcoding and MinION device (https://nanoporetech.com) (Table; Appendix Figure 1) (*4*). We classified sequence reads by using wf-metagenomics and wf-alignment in epi2melabs v.5.1.3 (Oxford Nanopore), mapped to the *F. tularensis* genome (GenBank accession no. NC_007880.1) by reference-based assembly using minimap2, and annotated using tbCon and ggplot in RStudio (Posit, https://posit.co) (Table). Subsequently, we tested lung and liver tissue from both animals for *F. tularensis* by immunohistochemistry and by culture and PCR using Centers for Disease Control and Prevention Laboratory Response Network proprietary protocols (Table). We then typed samples from positive animals by multilocus type sequencing of 6 genes (*fabH, tpiA, sdhA, rpoA,* *groEL,* and *pgm*) (*5*–*7*) and sequenced multiplexed amplicon libraries on the MiSeq platform (Illumina, https://www.illumina.com) (Table). We mapped amplicon sequence reads to reference genes from *F. tularensis* subsp. *holarctica* live vaccine strain, concatenated, and aligned with corresponding sequences from *F. tularensis* and other *Francisella* spp. to construct phylogenetic trees. 

**Table:** Tests performed on dead stranded beluga whales infected with *Francisella tularensis*, Cook Inlet, Alaska, USA*

Procedure	Method	Method detail	Laboratory	Manufacturer† or reference
Gross necropsy	NA	Tissues stored in whirl paks and swabs in cryovials with VTM or TSB with 15% glycerol and frozen at −80°C within 6 hours of sampling.	AVPS	Remel (VTM), Hardy Diagnostics (TSB)
Nanopore metagenomics	QiAMP DNA/RNA kit	Eluted in 50 μL elution buffer	UAA	QIAGEN
Nanopore metagenomics	Rapid SMART9N	Superscript IV TR-ase (Thermo Fisher Scientific†); primers RLB RT-9N, TTTTTCGTGCGCCGCTTCAACNNNNNNNNN and RLB TSO-RNA (r-hybrid oligo), GCTAATCATTGCTTTTTCGTGCGCCGCTTCAACATrGrGrG without DNase treatment. Cycling conditions: 42°C for 90 min; 70°C for 10 min; 4°C hold.	UAA	‡
Nanopore metagenomics	ONT† SQK-RPB114.96 kit (V14)	LongAmp Taq (2X; New England BioLabs†) PCR (95°C for 45 min, then 30 cycles at 95°C for 15 s; 56°C for 15 s; 65°C for 5 min; and 10 min final extension) with Rapid PCR barcoding using ONT SQK-RPB114.96 kit V14 on an ONT MinION Mk1B device running MinKNOW v.24.06.8 with high-accuracy basecalling.	UAA	(*4*)
Nanopore metagenomics: bioinformatics sequence read classification	wf-metagenomics and wf-alignment in Epi2me Labs v.5.1.3	NA	UAA	Epi2me Labs
Nanopore metagenomics: *F. tularensis* genome mapping	Reference-based assembly	Minimap2	UAA	NA
Nanopore metagenomics: sequence allignment and annotation	tbCon and ggplot in RStudio v. 2024.04.0+735	NA	UAA	NA
MLST: amplification	Amplification of 6 genes: *fabH, tpiA, sdhA, rpoA, groEL*, and *pgm*	Primers described in references	CDC	(*5*–*7*)
MLST: sequencing of amplicon libraries	Multiplexed amplicon sequencing on MiSeq platform	Nextera XT and V2 300 cycle reagent kit	CDC	Illumina
MLST: *F. tularensis* genome mapping	Reads were mapped to reference genes from *F. tularensis* subsp. *holarctica* LVS	CLC Genomics Workbench	CDC	QIAGEN
MLST: phylogenetic tree construction	Maximum-likelihood analysis	Generalized time-reversible nucleotide substitution model with gamma distribution (4 categories) plus invariant sites and 1,000 bootstrap replications	CDC	MEGAX
*F. tularensis* culture	LRN protocol	NA	CDC	CDC LRN
*F. tularensis* PCR	LRN protocol	NA	CSU VDL	CDC LRN
*Erysipelothrix* sp. PCR	NA	Primers ERy4423F and Ery4587R only	AVDL	(*8*)
Influenza A virus PCR	NA	Primers described in reference	Tufts and UAA	(*9*)
Histopathology	Hematoxylin and eosin staining	NA	HCS and AVPS	NA
Immunohistochemistry for *F. tularensis*	NA	NA	KSU	NA
Saxitoxin	ELISA	NA	WARRN West	Abraxis
Domoic acid	ELISA	NA	WARRN West	Abraxis

*AVDL, University of Georgia Athens Veterinary Diagnostic Laboratory; AVPS, Alaska Veterinary Pathology Services; CDC, Centers for Disease Control and Prevention; CSU VDL, Colorado State University Veterinary Diagnostic Laboratory; HCS, Histological Consulting Services; KSU, Kansas State University Veterinary Diagnostic Laboratory; LRN, CDC Laboratory Response Network; LVS, live vaccine strain; MLST, multilocus sequence typing; NA, not applicable; ONT, Oxford Nanopore Technologies; TSB, tryptic soy broth; Tufts, Tufts University; UAA, University of Alaska; VTM, viral transport medium; WARRN West, National Oceanic and Atmospheric Administration Wildlife Algal-toxin Research and Response Network West. †Manufacturers and products: Abraxis, https://www.bms.com; Epi2me Labs, https://epi2me.nanoporetech.com; Hardy Diagnostics, https://hardydiagnostics.com; MEGAX, https://www.megasoftware.net; Minimap2, https://github.com/lh3/minimap2; New England Biolabs, https://www.neb.com; Oxford Nanopore Technologies, https://nanoporetech.com; QIAGEN, https://www.qiagen.com; Remel, https://www.thermofisher.com; RStudio, https://posit.co; tbCon, https://github.com/jeremyButtler/bioTools; Thermo Fisher Scientific; https://www.thermofisher.com. ‡I.M. Claro et al., unpub. data, https://doi.org/10.12688/wellcomeopenres.17170.2. §GenBank accession no. NC_007880.1.

Both animals were pregnant adult females with markedly enlarged mediastinal lymph nodes, pleuritis, and pneumonia (Figure, panel A). One animal had severe multifocal random ecchymotic hemorrhage in the blubber (Figure, panel B). Histologic findings included necrosuppurative and histiocytic bronchopneumonia, lymphadenitis, and hepatitis (Appendix Figure 2, panels A–C). Immunohistochemistry demonstrated positive staining in areas of inflammation (Appendix Figure 2, panel D). Domoic acid and saxitoxin were not found, and PCRs and bacterial cultures yielded negative results or mixed organisms believed to be postmortem overgrowth (Appendix Table).

**Figure:**
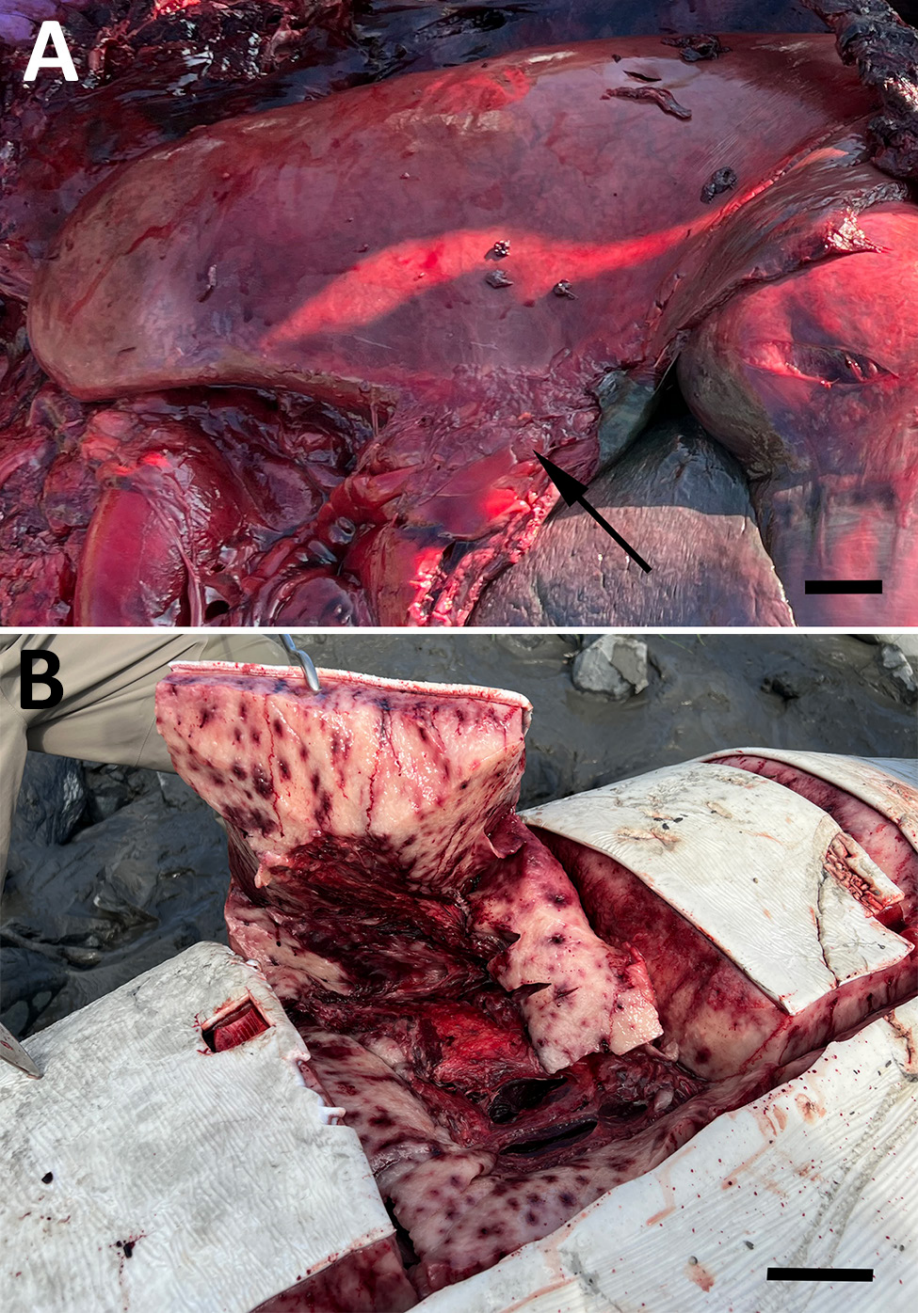
Gross examination of a beluga whale infected with *Francisella tularensis* subspecies *holarctica*, Cook Inlet, Alaska, USA. A) Lung and enlarged mediastinal lymph node (arrow). Scale bar = 6 cm. B) Ecchymoses in the blubber, showing extensive positive staining primarily in areas of inflammation. Scale bar = 5 cm.

We identified the causative organism by using metagenomics. We mapped sequence reads from animal 2023279 by reference-based assembly and found those reads to be distributed at low read depth (2–21×; 1,181 sequence reads; N50 = 275 nt, quality score = 9) across the 1.89-Mbp *F. tularensis* genome. We detected *F. tularensis* DNA in all samples by quantitative PCR with cycle threshold values <25. By multilocus sequence typing, we identified a concatenated sequence of 4,107 bp as *F. tularensis* subsp. *holarctica*. Phylogenetic analysis placed this strain in a clade identical to the 2023 pinniped case from Washington, as well as other isolates from the Northern Hemisphere (Appendix Figure 3).

Although Cook Inlet belugas are known to be susceptible to a variety of bacterial pathogens (*10*), *F. tularensis* has not been previously detected in this population, or in other cetaceans. The pattern of pathology represents the pulmonary form of tularemia, and the route of exposure was likely inhalation of contaminated water. *F. tularensis* is primarily a disease associated with freshwater, but the brackish nature of Cook Inlet and nearshore residence of belugas expose them to potentially contaminated freshwater runoff as well as to other reservoirs typically associated with freshwater (e.g., aquatic rodents, mosquito larvae) (*1*,*2*). The cause of the infections in a previously unreported host is unknown; however, host factors such as immunosuppression or environmental changes, such as increased runoff, could be considered. 

One human case of tularemia was reported in Cook Inlet’s largest adjacent city in 2023 (https://epi.alaska.gov/bulletins/docs/b2024_14.pdf); however, the circumstances of exposure were not reported. The propensity of whales to travel long distances could further disseminate this pathogen, increasing exposure to humans and wildlife. Our findings highlight a new risk to persons working in the marine environment and should be considered when assessing biosecurity and marine mammal health in the North Pacific.

Appendix Additional information for *Francisella tularensis* subspecies *holarctica* in stranded beluga whales, Cook Inlet, Alaska, USA.
