# Supplementary material for: Francisella tularensis Subspecies holarctica in Stranded Beluga Whales, Cook Inlet, Alaska, USA
Source: Emerg Infect Dis. 2025 Jun;31(6):1247–50. doi: 10.3201/eid3106.250033 (PMC12123914; doi:10.3201/eid3106.250033)
Supplement: Appendix — Additional information for Francisella tularensis subspecies holarctica in stranded beluga whales, Cook Inlet, Alaska, USA. [file 25-0033-Techapp-s1.pdf]

Article DOI: http://doi.org/10.3201/eid2806.250033

EID cannot ensure accessibility for supplementary materials supplied by authors. Readers who have difficulty accessing supplementary content should contact the authors for assistance.

Francisella tularensis Subspecies *holarctica* in Stranded Beluga Whales, Cook Inlet, Alaska, USA

Appendix

Appendix Table. Results of laboratory tests on dead stranded Cook Inlet beluga whales with *Francisella tularensis*

Animal ID	Tissue	Test	Laboratory*	Result
2023279	blowhole swab	aerobic culture	UCD	*Edwardsiella tarda*, *Enterococcus faecalis*, *Streptococcus* sp. *β hemolytic*
2023279	blowhole swab	influenza A virus PCR	Tufts	negative
2023279	mediastinal lymph node	influenza A virus PCR	UAA	negative
2023279	mediastinal lymph node	SARs CoV-2 PCR	UAA, AVDL	negative
2023279	fetal lung	*Francisella tularensis* PCR	CSU	negative
2023279	fetal lung	SARs CoV-2 PCR	CSU	negative
2023279	fetal spleen	aerobic culture	AVDL	Mixed growth including *Edwardsiella tarda*
2023279	fetal spleen, fetal lung, fetal liver	*Erysipelothrix* sp. PCR	AVDL	negative
2023279	heart	aerobic culture	UCD	*E. coli*, *Edwardsiella tarda*, *Enterococcus faecalis*
2023279	intestinal contents	Abraxis ELISA for saxitoxin	WARRN West	Below detectable limit
2023279	intestinal contents	Abraxis ELISA for domoic acid	WARRN West	Below detectable limit
2023279	liver	*Erysipelothrix* sp. PCR	AVDL	negative
2023279	liver	*Francisella tularensis PCR*	CSU	positive, Ct 23.04
2023279	liver	*Francisella tularensis* culture	CDC	negative
2023279	liver	Francisella MLST	CDC	*F. tularensis* subsp. *holarctica* positive
2023279	liver/lung pooled	*Francisella tularensis PCR*	CSU	positive, Ct 24.82
2023279	lung	*Erysipelothrix sp. PCR*	AVDL	negative
2023279	lung	*Francisella tularensis* PCR	CSU	positive, Ct 22.64
2023279	lung	*Francisella tularensis* culture	CDC	negative
2023279	lung	*Francisella* MLST	CDC	*F. tularensis* subsp. *holarctica* positive
2023279	lung	influenza virus PCR	UAA	negative
2023279	lung	SARs CoV-2 PCR	UAA, AVDL	negative
2023279	mediastinal lymph node	aerobic culture	UCD	*Edwardsiella tarda, Enterococcus faecalis, Streptococcus canis*
2023279	rectal swab	fecal pathogen culture	UCD	*Clostridium perfringens*
2023279	rectal swab	influenza A virus PCR and SARs CoV-2 PCR	UAA	negative
2023279	rectal swab	influenza A virus PCR, phocine distemper virus PCR, SARs CoV-2 PCR	Tufts	negative
2023279	spleen	aerobic culture	AVDL	*Edwardsiella tarda*
2023279	spleen	aerobic culture	AVDL	*Edwardsiella tarda*
2023288	blowhole swab	influenza A PCR	Tufts	negative
2023288	brain	aerobic culture	UCD	*Aeromonas* sp., *Edwardsiella tarda*
2023288	intestinal contents	Abraxis ELISA for domoic acid	WARRN West	Below detectable limit
2023288	intestinal contents	Abraxis ELISA for domoic acid	WARRN West	Below detectable limit
2023288	liver	*Francisella tularensis* PCR	CSU	positive, Ct 22.76
2023288	liver	*Francisella tularensis* culture	CDC	negative
2023288	liver	Francisella MLST	CDC	*F. tularensis* subsp. *holarctica* POS

Animal ID	Tissue	Test	Laboratory*	Result
2023288	lung	*Francisella tularensis* PCR	CSU	positive, Ct 21.84
2023288	lung	*Francisella tularensis* culture	CDC	negative
2023288	lung	Francisella MLST	CDC	*F. tularensis* subsp. *holarctica* POS
2023288	mammary gland	aerobic culture	UCD	*Aeromonas* sp. *Edwardsiella tarda*
2023288	mediastinal lymph node	aerobic culture	UCD	*Aeromonas* sp. *Edwardsiella tarda*, *Streptococcus bovis/equinus*
2023288	rectal swab	influenza A, SARS-CoV-2 and morbillivirus PCR	Tufts	negative
2023288	spleen	aerobic culture	UCD	*Aeromonas* sp. Edwardsiella tarda, *Clostridium perfringens*

*Athens Veterinary Diagnostic Laboratory (AVDL), Colorado State University Veterinary Diagnostic Laboratory (CSU), Tufts University Puryear laboratory (Tufts), University of Alaska Bortz laboratory (UAA), University of California Davis (UCD), NOAA Wildlife Algal-toxin Research and Response Network (WARRN West), Centers for Disease Control and Prevention Division of Vector-Borne Diseases (CDC).

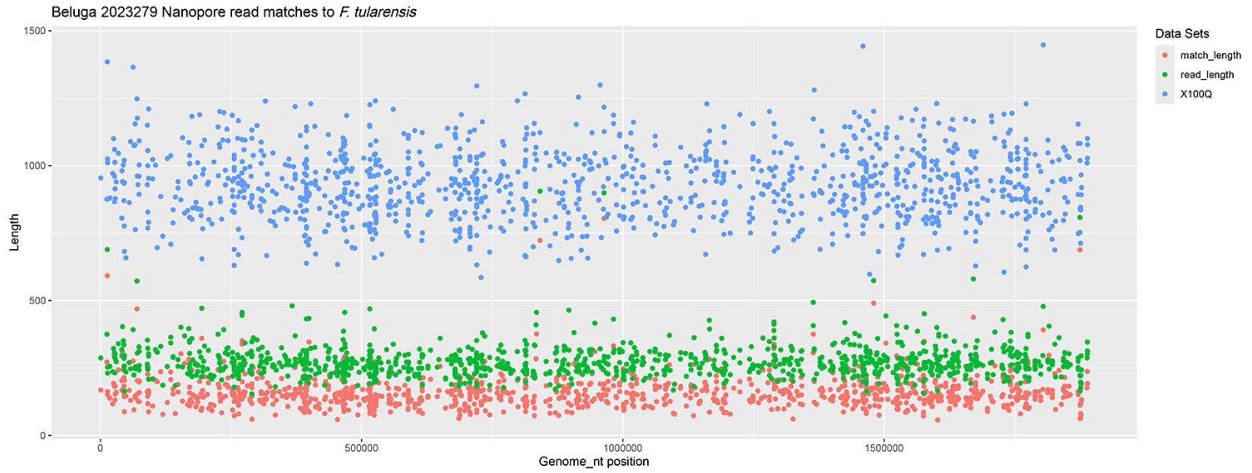

Appendix Figure 1. Reference based assembly and mapping of Smart9N nanopore reads distributed across *Francisella tularensis* genome (1.9Mbp; N = 1180 reads, N50 = 250nt, Average Q = 9). Green dot, read length; Red dot, matching nt length (trimming barcodes and adaptor sequence); Blue dots represent the 100X the Q score for each read rather than read length.

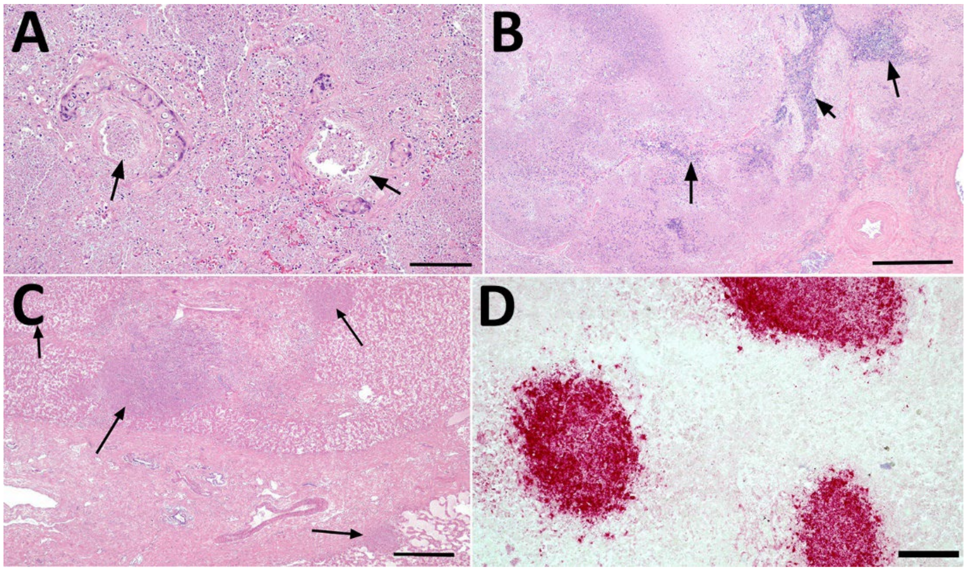

Appendix Figure 2. *Francisella tularensis* on histological (A–C) and IHC (D) examinations. A) Lung with a 150 µm bar. Arrows indicate bronchi surrounded by and filled with necrotizing histiocytic and suppurative inflammation; B) Mediastinal lymph node with a 500µm bar. Arrows indicate areas of remaining cortical lymphocytes; C) Liver with a 150µm bar. Arrows indicate areas of multifocal random inflammation; D) Immunohistochemistry for *F. tularensis* in liver with a 150µm bar. There is extensive positive staining primarily in areas of inflammation.

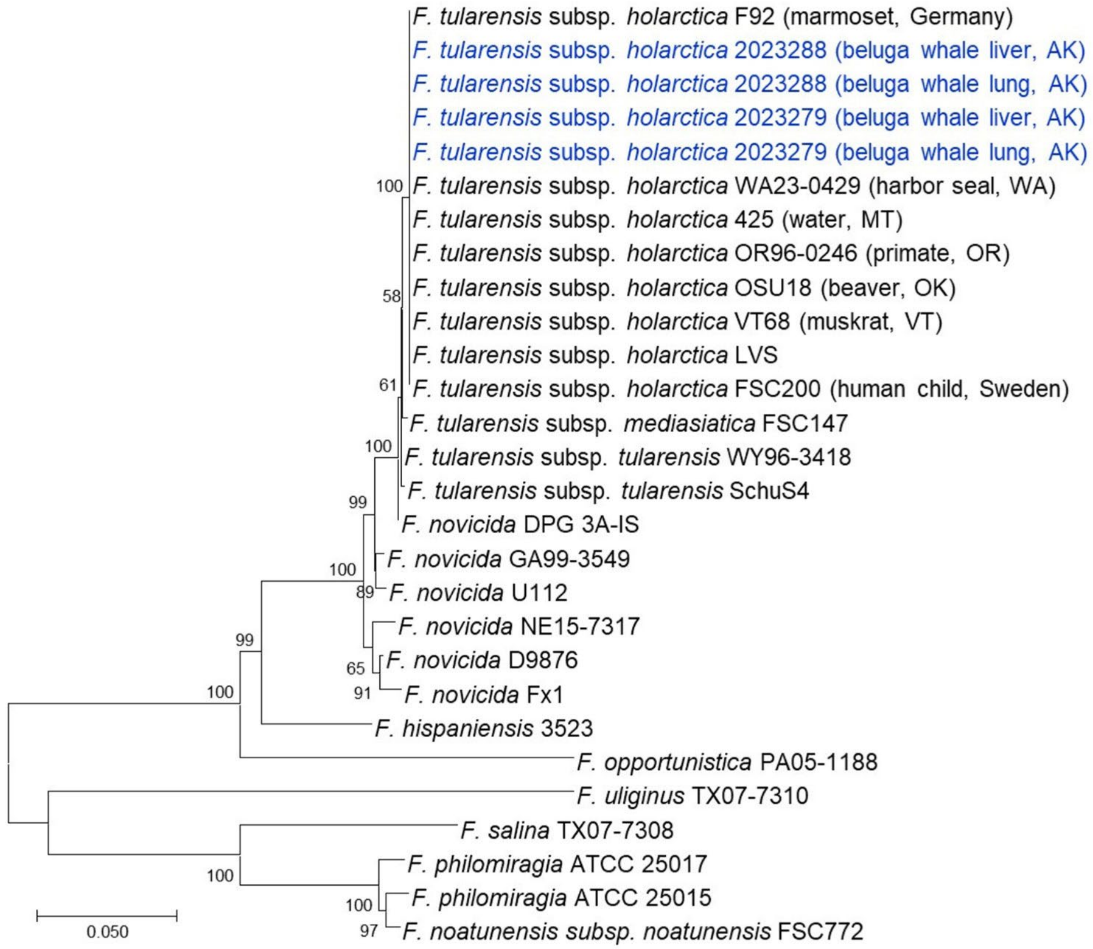

Appendix Figure 3. Genomics identification and typing of *Francisella tularensis* subsp. *holarctica* in stranded beluga whales. Maximum likelihood phylogeny of concatenated *F. tularensis* MLST sequences (comprising 6 genes, 4107bp total) from multiple tissue samples from stranded Beluga whales with reference *Francisella* spp. genomes, identifying *F. tularensis* subsp. *holarctica*.
